# Supplementary material for: Multi‐dataset identification of innovative feature genes and molecular mechanisms in keratoconus
Source: J Cell Mol Med. 2024 Sep 19;28(18):e70079. doi: 10.1111/jcmm.70079 (PMC11412914; doi:10.1111/jcmm.70079)
Supplement: Supplementary file 2 — Material S2. [file JCMM-28-e70079-s002.zip › 7.GSEA analysis/2.ATOH7/KEGG_TYROSINE_METABOLISM.html]

Details for gene set KEGG\_TYROSINE\_METABOLISM[GSEA]

|  || Dataset | ATOH7.group.cls#h\_versus\_l |
| Phenotype | group.cls#h\_versus\_l |
| Upregulated in class | h |
| GeneSet | KEGG\_TYROSINE\_METABOLISM |
| Enrichment Score (ES) | 0.47106668 |
| Normalized Enrichment Score (NES) | 1.5130327 |
| Nominal p-value | 0.016885553 |
| FDR q-value | 0.24526472 |
| FWER p-Value | 0.754 |
Table: GSEA Results Summary

  

Fig 1: Enrichment plot: KEGG\_TYROSINE\_METABOLISM      
 Profile of the Running ES Score & Positions of GeneSet Members on the Rank Ordered List

  

| SYMBOL | TITLE | RANK IN GENE LIST | RANK METRIC SCORE | RUNNING ES | CORE ENRICHMENT || 1 | TYRP1 | na | 148 | 0.726 | 0.0889 | Yes |
| 2 | TYR | na | 333 | 0.610 | 0.1620 | Yes |
| 3 | HPD | na | 607 | 0.519 | 0.2207 | Yes |
| 4 | HGD | na | 985 | 0.450 | 0.2676 | Yes |
| 5 | ADH1C | na | 992 | 0.448 | 0.3250 | Yes |
| 6 | ALDH1A3 | na | 1890 | 0.353 | 0.3444 | Yes |
| 7 | AOX1 | na | 2532 | 0.307 | 0.3653 | Yes |
| 8 | DCT | na | 2590 | 0.304 | 0.4026 | Yes |
| 9 | DBH | na | 2892 | 0.286 | 0.4307 | Yes |
| 10 | ADH1A | na | 3425 | 0.259 | 0.4486 | Yes |
| 11 | AOC3 | na | 3743 | 0.247 | 0.4711 | Yes |
| 12 | ALDH3B2 | na | 7831 | 0.122 | 0.3689 | No |
| 13 | METTL6 | na | 8529 | 0.108 | 0.3627 | No |
| 14 | ADH5 | na | 9514 | 0.090 | 0.3459 | No |
| 15 | ALDH3A1 | na | 10357 | 0.076 | 0.3313 | No |
| 16 | IL4I1 | na | 11846 | 0.055 | 0.2954 | No |
| 17 | MIF | na | 14343 | 0.027 | 0.2270 | No |
| 18 | TH | na | 15211 | 0.019 | 0.2045 | No |
| 19 | LCMT2 | na | 15459 | 0.017 | 0.1995 | No |
| 20 | LCMT1 | na | 16742 | 0.007 | 0.1635 | No |
| 21 | COMT | na | 16823 | 0.006 | 0.1619 | No |
| 22 | GOT1 | na | 17581 | -0.000 | 0.1402 | No |
| 23 | ADH7 | na | 18056 | -0.005 | 0.1271 | No |
| 24 | MAOA | na | 19096 | -0.014 | 0.0989 | No |
| 25 | GSTZ1 | na | 20598 | -0.028 | 0.0592 | No |
| 26 | FAH | na | 20792 | -0.030 | 0.0574 | No |
| 27 | METTL2B | na | 21293 | -0.035 | 0.0475 | No |
| 28 | ALDH3B1 | na | 22158 | -0.045 | 0.0283 | No |
| 29 | GOT2 | na | 23507 | -0.062 | -0.0026 | No |
| 30 | ADH1B | na | 25095 | -0.087 | -0.0372 | No |
| 31 | DDC | na | 25136 | -0.088 | -0.0271 | No |
| 32 | HEMK1 | na | 25901 | -0.102 | -0.0360 | No |
| 33 | TRMT11 | na | 26583 | -0.117 | -0.0407 | No |
| 34 | PNMT | na | 28767 | -0.172 | -0.0816 | No |
| 35 | ADH4 | na | 29617 | -0.198 | -0.0806 | No |
| 36 | ADH6 | na | 29876 | -0.207 | -0.0615 | No |
| 37 | TPO | na | 30642 | -0.234 | -0.0536 | No |
| 38 | MAOB | na | 32336 | -0.315 | -0.0619 | No |
| 39 | TAT | na | 33413 | -0.406 | -0.0408 | No |
| 40 | AOC2 | na | 34386 | -0.613 | 0.0099 | No |
Table: GSEA details [plain text format]

  

Fig 2: KEGG\_TYROSINE\_METABOLISM      
 Blue-Pink O' Gram in the Space of the Analyzed GeneSet

  

Fig 3: KEGG\_TYROSINE\_METABOLISM: Random ES distribution      
 Gene set null distribution of ES for **KEGG\_TYROSINE\_METABOLISM**
